# Can preoperative 25-hydroxyvitamin D levels predict transient hypocalcemia after total thyroidectomy?

**DOI:** 10.1007/s13304-021-01158-5

**Published:** 2021-09-26

**Authors:** Alberto Maria Saibene, Cecilia Rosso, Giovanni Felisati, Carlotta Pipolo, Simone De Leo, Paolo Lozza, Mario Gennaro Cozzolino, Loredana De Pasquale

**Affiliations:** 1 Department of Otolaryngology, Department of Health Sciences, Otolaryngology UnitASST Santi Paolo E CarloUniversità Degli Studi Di Milano, via di Rudinì 8, 20154 Milan, Italy; 2 Endocrino-Metabolic Department, Istituto Auxologico Italiano, Milan, Italy; 3 Department of Health Sciences, Nephrology Unit (Head: Professor Mario G. Cozzolino), ASST Santi Paolo E Carlo, Università Degli Studi Di Milano, Milan, Italy; 4 Thyroid and Parathyroid Surgery Service - Otolaryngology Unit (Head: Professor Giovanni Felisati), Department of Health Sciences, ASST Santi Paolo E Carlo, Università Degli Studi Di Milano, Milan, Italy

**Keywords:** Post-operative hypocalcemia, Post-operative hypoparathyroidism, Vitamin D, Parathyroid hormone, Total thyroidectomy complications

## Abstract

Transient postoperative hypocalcemia is a common complication after total thyroidectomy. Evidence on contributing metabolic factors is contradictory. Our work aims to define the role of preoperative 25-hydroxyvitaminD levels in developing transient postoperative hypocalcemia. 183 consecutive patients who underwent total thyroidectomy at our institution (May 2017–December 2019) were included in the retrospective study. We reported gender, age, estimated glomerular filtration rate, creatinine, preoperative 25-hydroxyvitaminD, serum pre- and postoperative calcium, pre- and postoperative PTH levels and transient postoperative hypocalcemia occurrences. We compared variables both among patients with and without transient postoperative hypocalcemia and between patients with different 25-hydroxyvitaminD levels (< 10 ng/ml deficitary; 11–30 ng/ml insufficient; > 30 ng/ml, normal). A binomial logistic regression model evaluating the risk for transient postoperative hypocalcemia was elaborated. Patients with transient postoperative hypocalcemia had lower levels of postoperative PTH (*p* < 0.001) and more frequently normal or deficitary 25-hydroxyvitaminD levels (*p* = 0.05). When comparing patients according to their 25-hydroxyvitaminD levels, insufficiency was associated with a lower rate of transient postoperative hypocalcemia (*p* = 0.05); deficiency was associated with higher preoperative PTH (*p* = 0.021), postoperative PTH (*p* = 0.043) and estimated glomerular filtration rate (*p* = 0.031) and lower serum creatinine (*p* = 0.014). In the regression model higher preoperative PTH (OR = 1.011, *p* = 0.041) and 25-hydroxyvitaminD deficiency (OR = 0.343, *p* = 0.011) significantly predicted transient postoperative hypocalcemia. Data analysis revealed a correlation between transient postoperative hypocalcemia and 25-hydroxyvitaminD levels: our work points towards the possibility to stratify the risk of transient postoperative hypocalcemia according to patients’ preoperative 25-hydroxyvitaminD status.

## Introduction

Postoperative hypocalcemia and hypoparathyroidism represent one of the most common complications of total thyroidectomy (TT) [1]. Hypocalcemia affects 30–50% of patients undergoing TT. Such a high rate led some authors to define post-thyroidectomy hypocalcemia a sequela, rather than a complication [2, 3]. Transient postoperative hypocalcemia (TPH) usually leads to longer hospitalization and/or recovery time, additional biochemical tests, potential symptomatic deficiencies and complications, with a direct increase of healthcare costs and disease burden [1].

Various factors may facilitate TPH, among them: hypothermia, prolonged surgical time, Graves’ Disease, female sex [1]. Parathyroid glands injury or accidental removal during TT is another established critical risk factor for TPH [4, 5]. Since these factors cannot explain each clinical scenario, other factors have been alternatively analyzed to assess their relationship with TPH. Already published studies correlate TPH with low early postoperative PTH levels and with calcium deficiency [6, 7]. The American Thyroid Association indicates low postoperative PTH as a predictor of post-total thyroidectomy hypocalcemia [8].

The association between TPH and preoperative 25-hydroxyvitamin D (25(OH)D levels, albeit widely studied, is less univocal. Some studies show an increased risk of postoperative hypocalcemia and a prolonged length of stay after TT in case of low preoperative 25(OH)D levels. Conversely, others state that 25(OH)D levels are not predictive of postoperative decrease in serum calcium [9–12]. Preliminary data indicate that calcium and 25(OH)D supplementation before TT may improve overall quality of life, shorten the hospital stay, and promote early discharge [13]. The role of pre-operative PTH levels in TPH is equally debated [14–16]. Most surprisingly, the role of serum creatinine and estimated glomerular filtration rate (EFGR) as indicator of chronic kidney disease have not been extensively studied in relationship with TPH. This despite the known mechanism that links chronic kidney disease, reduced renal activation of 25(OH)D into 1,25(OH)2D, reduced calcium absorption, and secondary PTH increase, a cycle which may indeed harbor an important role in TPH.

In this complex context, our work aims at evaluating the correlation between pre-operative factors and TPH risk in a retrospective cohort of patients undergoing TT at a single institution. More specifically, the authors aim at establishing a more definite connection between 25(OH)D levels and TPH, which may lead to future interventional strategies to decrease the incidence of this complication.

## Methods

### Study design

This study was conceived as a retrospective cohort study on all consecutive patients who underwent total thyroidectomy at a single institution (Santi Paolo e Carlo Hospital, Milan, Italy) from May 2017 to December 2019.

Given the retrospective nature of the study, it was granted an exemption from Internal Review Board approval, though it was conducted in full accordance with the Helsinki declaration. All patients included gave their informed consent to the surgical and medical procedures described in the study.

Inclusion criteria:

- Patients undergoing TT at our institution for any clinical indication
  Exclusion criteria:
- Patients who received preoperative supplementation therapy of Calcium and/or 25(OH)D
- Patients underwent total thyroidectomy with associated central neck dissection
- Patients affected with primary hyperparathyroidism
- Patients with accidental parathyroidectomy confirmed in the final surgical pathology report
- Patients with ongoing nephropathy
- Patients with definitive postoperative hypocalcemia

### Data collection

Medical records from 181 patients were considered eligible for the study.

For each patient we recorded information concerning sex, age at surgery, preoperative PTH levels, thyroid stimulating hormone (TSH) levels, serum calcium levels, serum creatinine levels, estimated glomerular filtration rate (EGFR), 25(OH)D levels, postoperative PTH and transient hypocalcemia during the hospital stay.

PTH was expressed as pg/ml (normal range 8.7–79.6 pg/ml). TSH was expressed as microIU/ml (normal range 0.47–4.68 microIU/ml). Serum calcium was expressed as mg/dL (normal range 8.4–10.2 mg/dL). Reported total calcium values were corrected for serum albumin levels. Serum creatinine was expressed as mg/dl (normal range 0.66–1.25 mg/dl). EGFR was calculated with the MDRD formula as described in Levy et al.[17]. 25-OH D was expressed in ng/ml. 25(OH)D levels lower than 10 ng/ml defined vitamin deficiency; 25(OH)D levels between 11 and 30 ng/ml defined vitamin insufficiency; 25(OH)D levels above 30 ng/ml were considered normal[18]. TPH was defined as serum calcium lower than 8.4 mg/dl on postoperative day one and two, recovering without need of calcium supplementation by 6 months.

### Statistical analysis and data availability

Comparison of binomial outcomes between groups was assessed with Pearson Chi Square. Mann–Whitney test and Kruskal–Wallis test were used for comparing variables between two and three groups, respectively. A Spearman’s Rho test was used to assess correlation between preoperative and postoperative PTH levels.

A multiple binomial logistic regression was ultimately carried out to elaborate a predictive model of TPH, based on preoperative variables, progressively removing non-statistically significant variables in an iterative fashion.

Data were analyzed on SPSS Version 25.0 (IBM Corporation. Armonk, NY. US). Statistic tests were two-sided, where available, and a *p* value ≤ 0.05 was considered statistically significant.

The data that support the findings of this study are available from S. Paolo Hospital’s database. Restrictions apply to the availability of these data, which were used under license for this study.

## Results

### Indications to total thyroidectomy, long-term complications and data retrieval

Clinical indications for total thyroidectomy included: goiter with normal thyroid function (52), hyperfunctional goiter (29), Graves’s disease (12), oncologically confirmed or suspicious thyroid nodules (88). Surgery was provided by the same first operator at the same Service of Thyroid and Parathyroid Surgery in the Unit of Otolaryngology. Histological results were: simple hyperplasia (12), nodular hyperplasia (81), Differentiated Thyroid Carcinoma DTC (86), papillary and medullary carcinoma (1) and hyalinizing trabecular tumor (1).

Sixty-four patients (35.36%) showed transient postoperative hypocalcemia.

Data recovered about gender; age at surgery; preoperative PTH, TSH, serum calcium, serum creatinine and 25(OH)D levels; EGFR; postoperative PTH levels; and transient hypocalcemia are reported in Table 1.

**Table 1:** Patient data according to postoperative transient hypocalcemia

Parameter	General population (*n* = 181)	Postoperative eucalcemia group (*n* = 117)	Transient hypocalcemia group (*n* = 64)	Statistical significance
Male to female ratio	50:131	34:83	16:48	*p* = 0.361*
Age at surgery	54 ± 24.5 (17–84)	53 ± 24 (20–84)	53 ± 23 (17–81)	*p* = 0.940**
Preoperative PTH	62.60 ± 35.05 (18.6–237.6)	59.30 ± 26.20 (18.6–237.6)	68.80 ± 42.23 (25.90–183.50)	*p* = 0.038**
Preoperative TSH	1.69 ± 2.02 (0.015–23.5)	1.7 ± 1.98 (0.015–23.5)	1.61 ± 1.9 (0.015–16.40)	*p* = 0.708**
Preoperative calcium	9.4 ± 0.4 (8.4–10.5)	9.5 ± 0.4 (8.6–10.5)	9.4 ± 0.38 (8.4–10.3)	*p* = 0.370**
Preoperative creatinine	0.8 ± 0.2 (0.4–1.9)	0.8 ± 0.3 (0.4–1.9)	0.7 ± 0.2 (0.6–1.6)	*p* = 0.937**
EGFR	87.53 ± 25.13 (33.67–159.21)	87.53 ± 25.94 (33.67–159.21)	87.47 ± 21.42 (6.39–149.99)	*p* = 0.696**
Preoperative 25(OH) VitaminD (ng/ml)				
< 10	45 (24.86%)	27 (23.08%)	18 (28.12%)	*p* = 0.045**
10–30	106 (58.56%)	76 (64.95%)	30 (46.88%)	
> 30	30 (16.58%)	14 (11.97%)	16 (25%)	
Postoperative PTH	33.8 ± 30.55 (3.4–180.9)	42.40 ± 25.10 (7.7–180.9)	15 ± 22.18 (3.4–95.8)	*p* < 0.001**
Postoperative calcium	8.1 ± 0.7 (6.4–9.5)	8.4 ± 0.2 (8.4–9.5)	7.6 ± 0.5 (6.4–8.3)	*p* < 0.001**

The table reports variables for the general study population, further broken down into the postoperative transient hypocalcemia group and for the postoperative eucalcemia group. The *p* value for each statistical comparison between the two groups is reported (*Pearson’s Chi square; **Mann–Whitney *U* test). Values are reported as ratio (*N*:*N*), absolute number (*N*(%)) or median ± interquartile range (minimum–maximum value). *EGFR* estimated glomerular filtration rate

Spearman’s Rho test showed a strongly significant correlation coefficient between preoperative and postoperative PTH (*p* = 0.001).

### Comparing patients with and without transient postoperative hypocalcemia

Patients with and without TPH were compared in terms of sex, age at surgery, preoperative PTH, preoperative TSH, preoperative calcium, preoperative serum creatinine, EGFR, preoperative 25(OH)D level, and postoperative PTH, and postoperative calcium with a either a Mann–Whitney U test (for nonparametric data) or a Pearson’s chi square (for binomial data). There were no significant differences between the two groups in terms of age (*p* = 0.940), preoperative TSH (*p* = 0.708), preoperative calcium (*p* = 0.37), preoperative serum creatinine (*p* = 0.937), EGFR (*p* = 0.696), and sex distribution (*p* = 0.361).

There was a statistically significant difference in preoperative PTH (*p* = 0.038), postoperative PTH (*p* < 0.001) and vitamin D levels (*p* = 0.045). Patients with TPH had lower levels of postoperative PTH and had a higher proportion of either completely normal or deficient vitamin D levels than patients without TPH.

### Comparing patients according to 25(OH)VitaminD levels

Patients from the three 25(OH)D level groups (deficiency, insufficient and normal) were compared in terms of all other evaluated variables with a either a Kuskal-Wallis test (for nonparametric data) or a Pearson’s chi square (for binomial data).

There was no difference in the three groups in terms of sex distribution (*p* = 0.491), age (*p* = 0.604), preoperative TSH (*p* = 0.676) and preoperative calcium level (*p* = 0.455). A statistically significant difference emerged in terms of preoperative PTH (*p* = 0.038), serum creatinine (*p* = 0.013), EGFR (*p* = 0.023), postoperative PTH (*p* = 0.029) and TPH rate (*p* = 0.045).

**Fig. 1:**
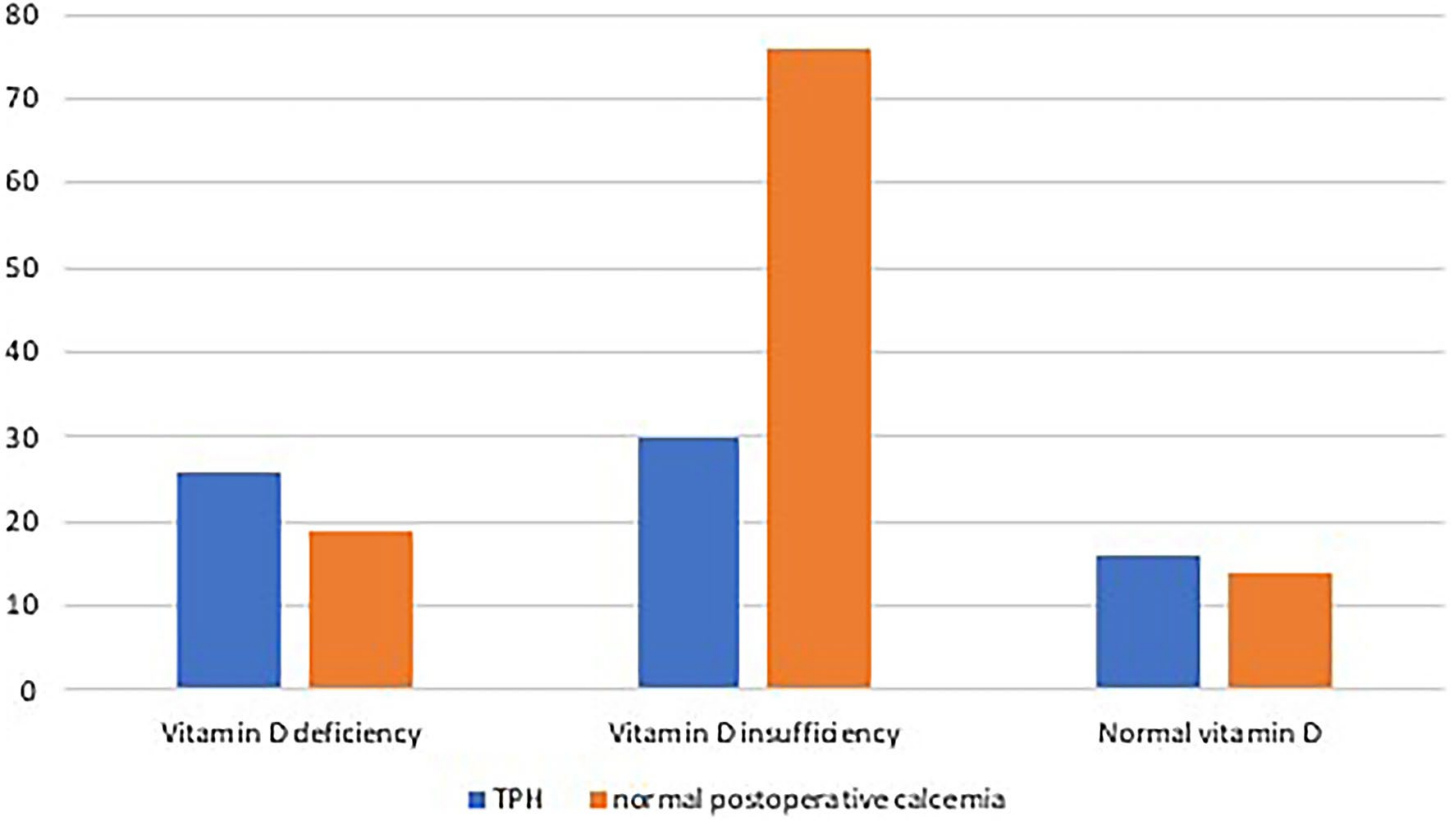
Column chart reporting the rate of patients with and without transient postoperative hypocalcemia according to their preoperative 25-hydroxyvitamin D levels

As reported in Table 2 the TPH rate difference between the three groups is remarkable (see also Fig. 1). Only 28.3% of patients with 25(OH)D insufficiency show TPH, significantly less than the other two groups (respectively 57,87% of patients with 25(OH)D deficiency and 53,33% of patients with normal levels of 25(OH)D). Also, as reported in Table 2, median preoperative and postoperative PTH and EGFR are higher and serum creatinine lower in patients with Vitamin D deficiency than in patients with Vitamin D insufficiency or normal vitamin D.

**Table 2:** Patients data according to preoperative vitamin D levels

Parameter	General population (*n* = 181)	Vitamin D deficiency (*n* = 45)	Vitamin D insufficiency (*n* = 106)	Normal vitamin D (*n* = 30)	Statistical significance
Male to female ratio	50:131	11:34	28:78	11:19	*p* = 0.491*
Age at surgery	54 ± 24.5 (17–84)	50 ± 26 (20–82)	55 ± 23 (20–81)	57 ± 19.25 (17–84)	*p* = 0.604**
Preoperative PTH	62.60 ± 35.05 (18.6–237.6)	79.60 ± 50.01 (24.8–237.6)	65.5 ± 31.225 (26.3–140.7)	51.75 ± 29.85 (18.6–129.2)	*p* = 0.019**
Preoperative TSH	1.69 ± 2.02 (0.015–23.5)	1.49 ± 1.45 (0.015–8.16)	1.84 ± 2.31 (0.015–23.5)	1.62 ± 1.9425 (0.27–16.4)	*p* = 0.676**
Preoperative calcium	9.4 ± 0.4 (8.4–10.5)	9.4 ± 0.3 (8.5–10.5)	9.5 ± 0.4 (8.6–10.4)	9.4 ± 0.35 (8.4–10.1)	*p* = 0.455**
Preoperative creatinine	0.8 ± 0.2 (0.4–1.9)	0.7 ± 0.2 (0.4–1.6)	0.8 ± 0.275 (0.5–1.9)	0.88 ± 0.3 (0.6–1.5)	*p* = 0.013**
EGFR	87.53 ± 25.13 (33.67–159.21)	92.22 ± 22.03 (44.16–159.21)	87.47 ± 25.635(42.12–134.8)	82.96 ± 16.345 (33.67–143.2)	*p* = 0.023**
Postoperative PTH	33.8 ± 30.55 (3.4–180.9)	40.8 ± 35.9 (3.4–120.6)	35.4 ± 27.05 (3.4–180.9)	27.70 ± 18.325 (3.4–85.4)	*p* = 0.029**
Postoperative calcium	8.1 ± 0.7 (6.4–9.5)	8.4 ± 0.7 (6.4–9.1)	8.4 ± 0.55 (6.7–9.3)	8.2 ± 0.725 (6.4–9.5)	*p* = 0.545**
Transient hypocalcemia					
No	117 (64.64%)	19 (42.22%)	76 (71.7%)	14 (46.67%)	*p* = 0.045*
Yes	64 (35.36%)	26 (57.78%)	30 (28.3%)	16 (53.33%)	

The table reports all patients’ variables in the general study population and subdivided according to the preoperative 25-hydroxyvitamin D levels. The *p* value for each statistical comparison between the two groups is reported (*Pearson’s Chi square; **Kruskal Wallis test). Values are reported as ratio (*N*:*N*), absolute number (N(%)) or median ± interquartile range (minimum–maximum value). *EGFR* estimated glomerular filtration rate

### Binomial regression

We elaborated a predictive model of TPH risk with a binomial regression, aimed at preoperative variables and patients’ characteristics. We iteratively removed from the regression all parameters with statistically non-significant *p* values (sex, *p* = 0.238; age, *p* = 0.418; preoperative TSH, *p* = 0.425; preoperative calcium, *p* = 0.471; and EGFR, *p* = 0.798), building a regression model including only preoperative PTH (*p* = 0.029, odd ratio 1.012) and vitamin D level (*p* = 0.029, odd ratio 0.703 for vitamin D insufficiency). The model showed good fit results (Hosmer and Lemeshow Test, chi square = 9.598; *p* = 0.294) and was able to correctly classify 64.4% of cases.

## Discussion

To the authors’ knowledge, this is the first literature report describing this complex, non-linear relationship between 25(OH)D levels and risk of TPH.

Our 35.36% TPH rate is consistent with the literature and corresponds to a significant patient group given the sheer number of TT performed on a daily basis worldwide [4, 5].

By excluding patients with incidental parathyroidectomy (whose literature rate ranges from 5 to 20%) we were able to focus our case series on other determinants for TPH, most eminently 25(OH)D levels [19].

By comparing patients with and without TPH in our case series, several significant differences emerged. Patients with TPH are more frequently characterized by either deficitary or normal 25(OH)D levels and lower postoperative PTH (Table 1).

Postoperative hypoparathyroidism is an already known determinant of TPH. Nevertheless 71,9% of TPH patients in our series did not show postoperative hypoparathyroidism. Therefore, we chose to further explore the other significant variable, i.e., 25(OH)D level.

Instead of exploring a linear relationship between TPH and 25(OH)D value, as already proposed by other authors with inconclusive results, we classified patients in three standard groups according to their 25(OH)D level, defined as deficiency (< 10 ng/ml), insufficiency (11–30 ng/ml) and normality (> 30 ng/ml) [9–12, 18]. This grouped analysis revealed an unprecedentedly known correlation between TPH risk and 25(OH)D levels. Analyzing variables in the three groups, significant differences emerged not only for 25(OH)D levels, but also for pre- and postoperative PTH, serum creatinine, EGFR and TPH rate (see Table 2). The different TPH risk between groups might be justified by the metabolic differences emerged between patients, with hypothetical mechanisms that we are exploring in the following paragraphs.

### 25(OH)D deficiency group

This group is characterized by higher pre- and postoperative PTH, lower serum creatinine and higher EGFR. This group has the highest rate of TPH (57.87%). Our hypothesis is that insufficient 25(OH)D levels might reduce dietary calcium absorption and determine a mild secondary hyperparathyroidism state.

The pathogenesis of secondary hyperparathyroidism is complex and can involve both hypocalcaemia and vitamin D deficiency and insufficiency. 25-hydroxyvitamin D is converted into the active form of vitamin D [1,25-dihydroxyvitamin (1,25D)] by the cytochrome P450 enzyme, 1-alpha-hydroxylase (CYP27B1) in the kidney and other tissues. Hence, low serum 25D concentrations contribute to reduced 1,25D levels by providing less substrate for conversion. 1,25D reduces plasma PTH levels through its interactions with the vitamin D receptor in the parathyroid glands, and concomitantly increases the intestinal absorption of calcium. Consequently, decreased serum 1,25D levels lead to reductions in serum calcium and excessive PTH secretion.

Several studies demonstrate a strong negative correlation between 25-hydroxyvitamin D and PTH, which appears clear not only in a severe state of 25-hydroxyvitamin D deficiency with probable secondary hyperparathyroidism, but also in a 25-hydroxyvitamin D insufficiency, where PTH serum levels gradually increase [20]. Sayed-Hassan et al. investigated the relationships between 25OHD, PTH, and bone mineral density in 156 participants. The group confirmed the negative correlation between 25-hydroxyvitamin D and PTH: more than third (35.3%) of the case series had secondary hyperparathyroidism, with significant higher prevalence among participants in the lowest 25-hydroxyvitamin D quartile compared to those in the highest quartile [21].

PTH increase compensates 25(OH)D deficiency inducing bone resorption and calcium release. Following postoperative PTH reduction (though without significant rates of overt postoperative hypoparathyroidism), these patients' skeletal system is immediately allowed to retain more calcium. Indeed, 19 out of 45 patients in this group showed postoperative hypocalcemia, with a *hungry bone disease*-like mechanism. They might thus mimic the already known hungry bone disease that characterizes patients surgically treated for primary hyperparathyroidism [22, 23].

### 25(OH)D insufficiency group

This group shows intermediate preoperative PTH, serum creatinine and EGFR levels and the lowest TPH rate (28,3%). Though free from the tendency to secondary hyperparathyroidism characterizing patients with 25(OH)D deficiency, these patients show a moderate parathyroid hyperfunction. Our hypothesis is that these patients’ calcium homeostasis is in a steady state, prone to continuous compensation. When PTH levels decrease after TT, serum calcium stability is provided by an appropriated bone mineralization and a flexible slight hyperfunction of parathyroid glands.

### Normal 25(OH)2 group

The smallest group in our series is characterized by the lowest levels of pre- and postoperative PTH, lowest EGFR and highest serum creatinine. These patients show a significant prevalence of TPH (53.33%), only slightly lower than in patients with deficitary 25(OH)D. We hypothesize in these patients a less adaptive calcium homeostasis. In this patient group, the postoperative decrease of PTH due to thyroid neck compartment manipulation might not be buffered as quickly as in patients with insufficient 25(OH)D, thus facilitating TPH.

The identification of these three different metabolic groups might explain the conflicting literature reports about the role of 25(OH)D in TPH [9–12]. The TPH rate appears highest at the end of the 25(OH)D values spectrum. Such distribution might have hampered correlations on less conspicuous case series. Our observations are reinforced by the strong correlation emerging between pre- and postoperative PTH levels. The well-known postsurgical PTH drop is the least capable of explaining TPH in patients with higher preoperative levels of PTH. This observation calls for further metabolic motivations for TPH, which might be found in the previously expressed hypothesis.

Our binomial regression on the TPH risk further reinforces our observations. In this solid—though preliminary—predictive model, PTH levels and 25(OH)D levels allow identifying patients at risk for TPH. On the other hand, the regression suggests that serum creatinine and EGFR do not have a significant role in TPH risk. The different distribution of these values in the 3 25(OH)D groups might nevertheless have a role in other metabolic processes which are still open to exploration.

It has to be noted that our study has a major design limitation: the retrospective design. It limits the value of the study, despite our effort in minimizing biases and confounding factors and selecting a significant continuous patient series. Our observations would be undoubtedly strengthened by a prospective clinical trial, taking into account the same preoperative parameters and, possibly, providing preoperative therapy for patients at higher risk.

Our data indeed strengthen the need for a prospective clinical trial, investigating targeted preoperative 25(OH)D and/or calcium supplementation in the specific group of patients with 25(OH)D deficiency. Such supplementation might mitigate the hypothesized secondary hyperparathyroidism, reducing the TPH risk.

Our primary hypothesis is that the routine supplementation of calcium and/or 25(OH)D already scrutinized by other authors could become more effective if targeted, according to 25(OH)D and PTH status [24]. On the base of the result of our study of a greater risk of TPH in pre-operative deficiency and normal 25(OH)D groups than in insufficiency, only patients with pre-operative 25(OH)D deficiency and high PTH levels might benefit from mild preoperative 25(OH)D supplementation, in order to bring 25(OH)D from a deficiency to insufficiency, but not normal level. Pre-operative normal 25(OH)D/low PTH patients might require only calcium supplementation. Grzegory et al*.* reviewed 15 studies about preoperative calcium and 25(OH)D supplementation in patients undergoing TT [13]. Supplementation effectiveness was compared to no prophylaxis in 10 studies. The review showed a clinical and economic advantage for routine perioperative prophylactic supplementation of 25(OH)D and/or calcium as compared to no prophylaxis. Two of the reviewed studies were placebo-controlled and showed a significant reduction of TPH incidence in patients who received a preoperative supplementation of Calcium Carbonate or 25(OH)D [25, 26]. Given these literature data and our novel findings, it is our strong conviction that further studies in these regards will allow a better selection of patients at risk of TPH and optimize the cost-effectiveness of preoperative supplementation.

## Conclusions

Our study revealed a correlation between transient postoperative hypocalcemia and 25-hydroxyvitaminD levels, hypothetically related to the mechanisms of calcium metabolism. Our work points towards the possibility to stratify the risk of transient postoperative hypocalcemia according to patients’ preoperative 25-hydroxyvitaminD status and suggests the need for further research towards targeted 25-hydroxyvitaminD and calcium supplementation.

## Data Availability

The datasets generated during and/or analysed during the current study are available from the corresponding author on reasonable request.
